# Giant Cell Angiofibroma in Unusual Localization: A Case Report

**DOI:** 10.1155/2012/408575

**Published:** 2012-04-18

**Authors:** Emel Ebru Pala, Rafet Beyhan, Umit Bayol, Suheyla Cumurcu, Ulku Kucuk

**Affiliations:** Pathology Department, Tepecik Training and Research Hospital, Yenisehir, Izmir, Turkey

## Abstract

Giant cell angiofibroma (GCA) was initially described as a potentially recurrent tumor in the orbit of adults. However, it is now recognized that it can also present in other locations. The morphological hallmark is a richly vascularized patternless spindle cell proliferation containing pseudovascular spaces and floret like multinucleate giant cells. Our case was a 32-years-old female complaining of painless solitary nodule arising on the occipital region of the scalp, which was diagnosed as giant cell angiofibroma. We report the case because of its extremely rare localization.

## 1. Introduction

A giant cell rich form of hemangiopericytomas-solitary fibrous tumors (HPC-SFT) was described by Dei Tos as giant cell angiofibroma (GCA) [1]. Although originally identified in the orbital region, this tumor may occur in diverse locations. GCAs are well-circumscribed variably encapsulated, small (median, 3 cm) lesions. Extraorbital tumors tend to be larger than orbital tumors. Haemorrhagic and cystic changes may be observed on the cut section. It shows histologic appearances intermediate between solitary fibrous tumor and giant cell fibroblastoma of soft tissue. It is characterized by the presence of cellular and sclerosing areas, keloidal collagen deposition, thick-walled vessels, and multinucleated giant stromal cells often lining pseudovascular spaces. Mitotic activity ranged from 1 to 3 mitoses/10 high-power fields. Treatment of GCA consists of simple tumorectomy.

## 2. Case Report

**Figure 1:**
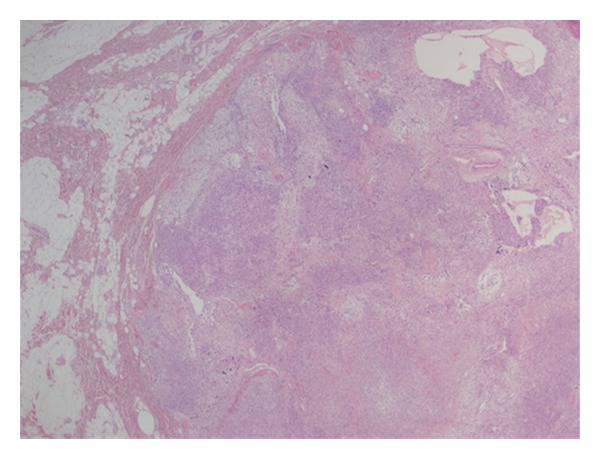
Well circumscribed subcutaneous tumor mass (HE, ×40).

**Figure 2:**
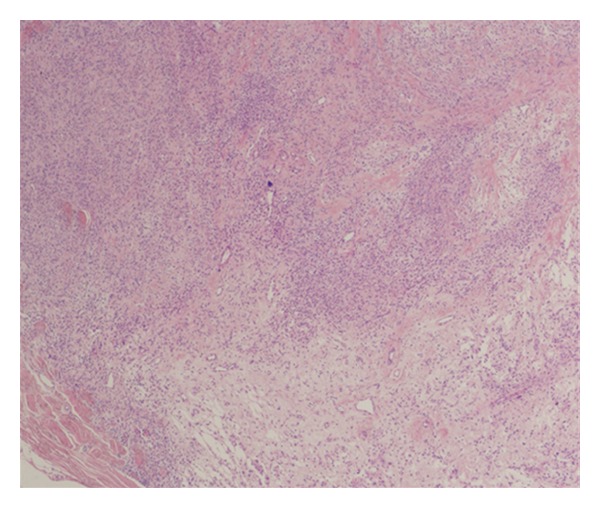
Admixture of hypocellular and hypercellular areas (HE, ×100).

**Figure 3:**
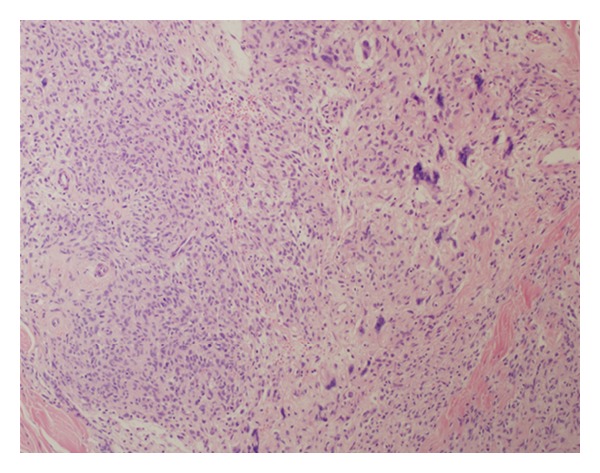
Patternless giant cell and spindle cell proliferation (HE, ×100).

**Figure 4:**
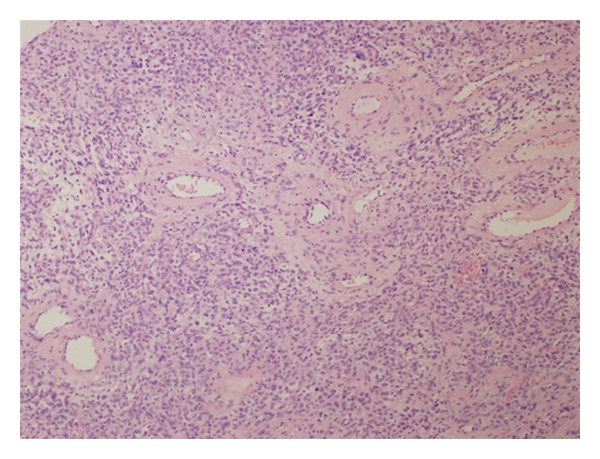
Prominent perivascular hyalinization (HE, ×100).

**Figure 5:**
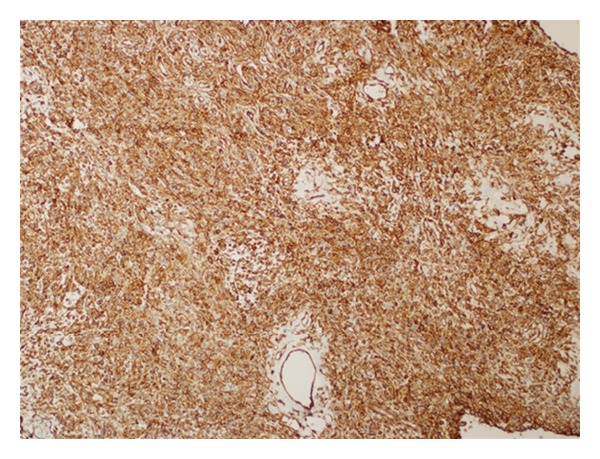
Diffuse CD34 positivity in tumor cells (DAB, ×100).

**Figure 6:**
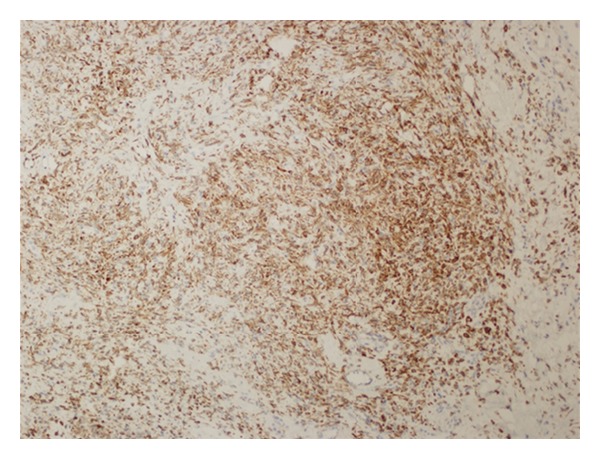
Bcl2 positivity in tumor cells (DAB, ×100).

We present a 32-year-old female complaining of painless solitary nodule arising on the occipital region of the scalp. When she first noticed the nodule two years ago, it was 13 mm. After 4 months the size of the nodule became 17 mm. A year later, superficial soft tissue ultrasonography revealed a 24 × 10 mm solid subcutaneous mass with hyperechogenic, heterogeneous density. And it was totally excised by a plastic surgeon. Her medical history did not show any distinctive features. On macroscopic examination, red-brown, 25 × 15 × 6 mm mass showed well-defined margins. Microscopic examination revealed a subcutaneous well-circumscribed mass, consisting of both cellular areas with especially oval-round-to-spindle-shaped cells intermixed with floret like giant cells in a patternless pattern and hypocellular areas together (Figures 1, 2, and 3). Giant cells often lined the pseudovascular spaces. The lesion was highly vascularized with varying caliber of blood vessels and involved perivascular hyalinization (Figure 4). Also we noticed subcapsular hemorrhage due to the surgical procedure. There was a small group of mononuclear inflammatory cells around pseudovascular spaces. The cells had vesicular chromatin, indistinct nucleoli, and eosinophilic cytoplasm with indistinct cell borders. Necrosis and cytologic atypia were absent. The number of mitotic figures was 2 per 10 HPFs. The cells were not encircled by reticulin fibers. Immunohistochemistry showed strong CD34 (Figure 5), bcl-2 (Figure 6), vimentin reactivity and focal CD99 positivity. S100, Desmin, SMA, CD117, CD31, and CD68 were negative. Ki 67 proliferation index was 2%.

## 3. Discussion

GCA is a condition that represents the transitional stages within the HPC/SFT spectrum. It is accepted as a variant of HPC/SFT. It displays all features of a classic SFT but is identified by pseudovascular spaces lined by multinucleated stromal giant cells. HPC, SFT, giant cell fibroblastoma (GCF), and GCA are CD34 (+) fibroblastic soft tissue tumors. And these tumors have a spectrum of overlapping morphologic and immunophenotypic findings. GCA is a slowly growing soft tissue mass. Although it is a distinctive orbital soft tissue tumor, it can also present in other locations, including head and neck, back, retroperitoneum, hip, vulva, axillary-inguinal regions, posterior mediastinum, and oral cavity. Microscopic examination reveals cellular spindle cell proliferation between hyalinized blood vessels and scattered multinucleated floret like giant cells. Mononuclear and multinucleated stromal cells are characteristically positive for vimentin, CD34, CD99 and, less frequently bcl-2, but negative for muscle specific actin, desmin, CD31, CD117, S100. GCF is one of the main entity in the differential diagnosis of GCA. GCF was first described in 1982 as a juvenile form of dermatofibrosarcoma protuberans (DFSP) [2]. GCF and DFSP show same translocation. GCF develops as a painless nodule in the dermis/subcutis. It mainly affects infants and children (median age: 3). They are ill-defined, infiltrative masses which composed of hypocellular areas with spindle/giant cells also DFSP-like storiform areas. Most of the GCFs express CD34. Recurrences have often developed but metastases have not been reported [3]. We should also think of fibrous histiocytoma (FH) in the differential diagnosis. But FHs are usually solitary dermal nodules with irregular penetration of the subcutis at the deep border. And the overlying epidermis frequently shows some degree of hyperplasia. In contrast to GCA, CD34 is absent within lesional cells of FH. As our case was a young adult and had a well-defined CD34 (+) tumor without any atypia, our diagnosis was GCA.

Furusato et al. [4] reviewed and analyzed 41 fibroblastic orbital tumors which originally were diagnosed as hemangiopericytomas (16/41), fibrous histiocytomas (9/41), mixed hemangiopericytoma/fibrous histiocytoma (14/41), and giant cell angiofibroma (2/41) of orbit. After histologic and immunohistochemical (CD34, CD99, bcl2, Ki67, p53) review, all cases were reclassified as solitary fibrous tumor. The results of this study suggested that these entities share overlapping morphologic and immunohistochemical features and should be designated as solitary fibrous tumors.

Sonobe et al. [5] detected abnormalities of chromosome 6 with a common pattern involving 6q13 together with other chromosomal aberrations in a typical case of giant cell angiofibroma. This was the first reported case of giant cell angiofibroma demonstrating chromosomal abnormalities. Qian et al. [6] reported a case of GCA in the neck whose chromosome analysis showed a karyotype with t(12;17)(q15;q23), del(18)(q21) in all 20 cells analyzed. This was the second case of GCA with chromosomal aberrations and the first case of GCA with t(12;17) occurring as an extraorbital mass. This cytogenetic abnormality in GCA is different from the t(17;22)(q22;q13) found in related lesions, such as giant cell fibroblastoma and solitary fibrous tumor, none of which has a specific chromosomal abnormality.

There are nearly 25 cases of extraorbital GCA in the literature [7–10]. Thomas et al. [8] reported four extraorbital GCA cases: two in the groin, one in axillary soft tissue, and one in parotid. Electron microscopy showed fibroblastic features in all cases and schwannian features in one case. All of the patients were well without recurrent disease on followup.

Besides the rarity, GCA should be kept in mind when the histopathologic examination reveals a well-defined fibroblastic patternless, CD34 (+) subcutaneous tumor mass.
